# Activation, Steady-State and Passivation Regimes for Ethene Hydrogenation over a Pd/Al**_2_** O**_3_** Catalyst: An *Operando* Neutron Imaging Study

**DOI:** 10.1021/acs.jpcc.6c01622

**Published:** 2026-04-22

**Authors:** Hamish Cavaye, Christos Ballas, Asma Nadia, Winfried Kockelmann, Stewart F. Parker, Paul Collier, Andrew P.E. York, David Lennon

**Affiliations:** † 97008 ISIS Pulsed Neutron and Muon Source, STFC Rutherford Appleton Laboratory, Chilton OX11 0QX, U.K.; ‡ School of Chemistry, University of Glasgow, Glasgow G128QQ, U.K.; § 263220 Johnson Matthey Technology Centre, Blounts Court, Sonning Common, Reading RG4 9NH, U.K.

## Abstract

Neutron imaging combined with in-line mass spectrometry is used to perform an *operando* study of hydrogen adsorption and ethene hydrogenation over a powdered 5 wt % Pd/Al_2_O_3_ catalyst contained within a stainless-steel reactor. The approach adopted enables the partitioning of hydrogen throughout the catalyst bed to be examined as reaction conditions are varied. Aspects of the catalyst activation procedure are examined including drying and reduction stages. Spatially resolved temporal profiles indicate how hydrogen is partitioning throughout the length of the reactor during these catalyst pretreatments. For the case of hydrogen exposure to the as-received catalyst at 293 K, while the mass spectrometer detects hydrogen breakthrough after 20 min, the neutron intensity profiles for 5 spatially distinct regions along the catalyst bed map out the progression of hydrogen along the length of the bed throughout and beyond the 20-min period, allowing the spatially resolved varying rates of adsorption to be assessed, and showing that hydrogen adsorption is fastest at the top of the catalyst bed. These neutron imaging profiles are discussed in terms of coincident reduction and drying events. Addressing matters of reaction engineering, the ethene hydrogenation experiments performed at 333 K examine how hydrogen supply affects the reactor/catalyst combination. Operational conditions are varied from hydrogen-excess to hydrogen-lean regimes. Hydrogen starvation experiments are used to assess the durability of the activated catalyst. The complete recovery of catalytic activity following reinstatement of hydrogen supply following a period of ethene-only feed demonstrates the reversibility of the hydrogenation process over this catalyst. The operando neutron imaging approach outlined here provides a new perspective on how hydrogenous entities pass through an extended catalyst bed.

## Graphical abstract

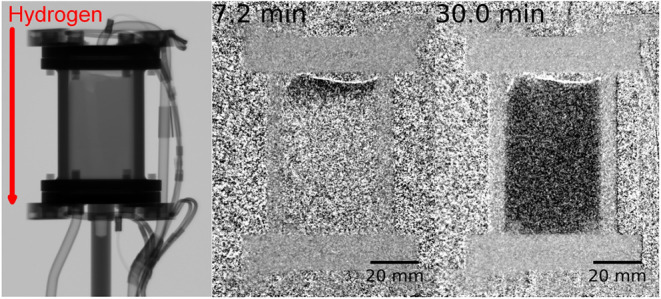

## 1 Introduction

For a given set of reaction conditions, outcomes from a heterogeneously catalyzed reaction are invariability critically dependent, first, on the catalyst and, second, on the reactor that contains the reaction/catalyst system. This awareness then defines a driver to investigate the catalyst while it is operating within a stated reactor configuration, a topic that comes under the scope of reaction engineering.^1^ One approach to investigate such complex reaction mediums is to use imaging techniques to explore how the catalyst is functioning during a reaction. X-ray imaging methods are becoming increasingly established, which are particularly relevant in examining metal-based catalysts.^2^ This scenario then provides the opportunity to evaluate structural parameters and changes in oxidation states of the catalyst within a reactor. Limitations to this method applied to a typical organic transformation include an inability of X-ray techniques to probe the reagents/products and, typically, the reactor used in such operations is a glass capillary or quartz reactor; something a long way away from reactors used in industrial operations, which tend to be made of steel, invariably stainless steel. The use of quartz is not inherently problematic; however, it cannot endure the high temperatures and pressures that are used in many real-world applications and can be more difficult to machine. Another option in the imaging armory is the technique of magnetic resonance imaging (MRI).^3^ In contrast to the X-ray imaging option, MRI can provide important chemical information on reagent-to-product transformations. In ideal circumstances, the method can be quantitative.^4^ Although, as in the X-ray case, glass reactors are favored with steel vessels being inappropriate for use within the magnetic field used with this technique. Adopting the goal of the imaging techniques to determine chemical composition as one traverses along the catalyst bed, an alternative approach is to physically sample the reaction mixture at different points along the length of the reactor. For example, the Spaci-mass spectrometry system adopts this approach, which is ideally set up to examine reaction fronts within steel reactors.^5^ However, in this methodology a large drawback is the need to insert an invasive probe within the reactor, potentially altering the reaction system behavior observed.^6^

In addition to these established methods of probing catalyst/reactor combinations, the recent awareness that neutron imaging procedures could be applied to catalytic systems is proving to be a fruitful area of endeavor. Pioneering the vanguard are the group of Andreas Borgschulte using the cold neutron beamline ICON25, located at the Swiss Neutron Spallation Source of the Paul Scherrer Institute (PSI) in Switzerland.^7–9^ Early work from this group used neutron imaging to examine hydrogen in Cu/ZnO catalysts during methanol synthesis.^10^ Further work used neutron imaging to distinguish irreversible hydrogen surface adsorption from reversible bulk absorption on a range of catalysts.^11^ The group have also highlighted the benefits of expanding the technique to *operando* neutron imaging.^12^ Recently, they have examined the hydrogenation of carbon dioxide to make methane, where analysis of the water byproduct was used to define the spatial and temporal conversion of the processes. The work was supplemented by modeling that was used to propose optimum reactor dimensions for the reaction conditions studied.^13^

Neutron imaging investigations of heterogeneously catalyzed reactions have also been performed by Cavaye and coworkers using the IMAT instrument of the ISIS facility that is located at the Rutherford Appleton Laboratory in the UK. In the first instance, *in situ* real-time neutron imaging of H_2_ adsorption and D_2_ exchange on carbon-supported Pd catalysts was undertaken using a combination of aluminum and stainless-steel reactors.^14^ Subsequently, the technique was applied to investigate the hydrogenation of ethene over a 5 wt % Pd/C powder catalyst located in a fixed bed stainless steel reactor.^15^ Modulations of the incident gas stream led to the observation of spatially resolvable fronts moving across the catalyst bed and illustrated the transport and diffusion of reagents from the reactor inlet across to the reactor exit.

The current work adopts the *operando* concepts outlined by Borgschulte and coworkers^9^ to further examine the hydrogenation of ethene; this time over a 5 wt % Pd/Al_2_O_3_ powder catalyst and with a mass spectrometer to analyze the reactor exit gas. Via a series of discrete experiments, spatial and temporal aspects of elementary processes linked to this model hydrogenation reaction are examined. Initial catalyst treatment in terms of hydrogen adsorption and reduction of the Pd particles shows a role for water production and a concomitant drying process throughout the catalyst bed. Quantification of the hydrogen uptake is undertaken by a combination of mass spectrometer-derived breakthrough measurements and neutron imaging data. Ethene hydrogenation is studied as a function of hydrogen concentration, with contrasting mass spectrometer and neutron imaging profiles providing information on hydrogen transport rates across the length of the reactor. Finally, the reversibility of the hydrogenation reaction is explored by examining ethane production after the catalyst had experienced a period of hydrogen starvation. In this way, the *operando* approach of combining neutron imaging capability with analysis of the reactor exit gas is revealing how hydrogenous moieties are partitioned throughout the length of the reactor in ethene hydrogenation over Pd/Al_2_O_3_. Crucially the presence of these concentration gradients is not necessarily apparent simply by inspection of the composition of the reactor exit gas.

## 2 Experimental Section

### 2.1 Sample Preparation and Reaction Conditions

29.2 g of a 5 wt % Pd/γ-Alumina powder catalyst (Fisher Sci., Cat. # 011713) was loaded into a flat stainless-steel cell (dimensions of sample-relevant part approximately 40 w x 65 h x 13 d mm). For temperature control, the top and bottom flanges of the cell were each fitted with two electrical cartridge heaters (WATLOW 1/8 Firerod, 80 V, 80 W) in parallel and with a temperature sensor. The base of the cell was lined with quartz wool (Elemental Microanalysis) then the catalyst loaded into the cell. As part of the catalyst charging process, the reactor was regularly tapped and the catalyst pressed with a spatula, so that the bed was evenly compressed. A layer of quartz wool was then packed in at the top of the bed and the reactor sealed. The cell was equipped with Swagelok fittings to connect to the external pipework, allowing gases to enter through the top of the cell, flow through the sample, and exit at the bottom (as viewed in the radiographs). It is noted that the Al_2_O_3_ support will contain a non-negligible amount of hydroxyl groups on the surface, which likely lead to some level of neutron attenuation during the experiment. However, within the temperature ranges used in this study, these hydroxyl groups are likely to remain invariant and can be considered part of the background of the neutron radiography data, and are therefore not considered further.

During beamtime operation, the cell was connected to a gas handling panel through approximately 6 m of 1/8” diameter stainless-steel line. The exit line of the reactor was connected to an extraction system and a Hiden Analytical mass spectrometer for exhaust gas analysis. The panel was located outside the IMAT experimental beam area and equipped with the following gases: He (Air Liquide, 99.999%), H_2_ (Compressed Gas Solutions Ltd., 99.9995%), and ethene (BOC, 99.9%). Gas flow rates were individually controlled through a series of HFC-302 Teledyne mass flow controllers connected to Chell CCD100 Controller boxes. The time scale for gas switching events was 1–2 s at the gas manifold but the time taken for such an event to be registered at the exit line for an empty cell was approximately 2 min. A schematic of the gas flow can be seen in Figure S1.

### 2.2 Neutron Radiography

Neutron radiography was performed at the IMAT beamline,^16^ using a polychromatic neutron beam of wavelength range 0.7–6.7 Å. The sample cell was mounted approximately 45 mm in front of a ZnS/LiF:Cu scintillator screen (200 μm thickness) of a neutron camera box. The scintillator screen was at a distance L = 10.4 m from a beam aperture of diameter D. The scintillator was coupled via a 45-degree mirror and a focusing lens of 50 mm focal length (Fstop 1.2) to a CMOS camera (ANDOR Zyla sCMOS 4.2, Oxford Instruments, UK) with 2048 × 2048 pixels, providing a field-of-view of 211 × 211 mm^2^ and a pixel size of 103 × 103 μm^2^. With a beam aperture of D = 80 mm and an L/D ratio of about 130, the geometric blur and resolution limit was about 345 μm.

Radiographs were obtained using looping exposures of 5 s each, with approximately 1–2 s of overhead time for data transfer between frames. Consequently, the temporal resolution of the data was one frame every ∼ 6–7 s.

### 2.3 Data Analysis

Within the frame workup procedure, the following calibration workflow was adopted using custom-written Python scripts: the detector output images first underwent removal of bright and dark outliers before open beam (flat field) and dark calibration operations were applied; a direct beam region of interest (RoI) normalization was applied to correct for variations in beam intensity that exist within a run (indicated in Figure 1, gray rectangle); data were then saved to disk. To follow how a particular perturbation (e.g., change in gas composition at time t) affected the reactor image, a median average frame was created from the first 20 frames of a run, prior to any perturbation being imparted, and saved as a reference image. In this way, changes in the image as a function of time-on-stream are referenced to this averaged value (normalized intensity, I_0_ = 1.000 au). This process of normalization was necessary due to large absolute gray value differences in each RoI being investigated, e.g., the gray values for the air regions outside of the reactor were substantially larger than for those inside the reactor. Normalization in this manner allowed the relative changes in intensity during any perturbation to be compared across each RoI.

**Figure 1:**
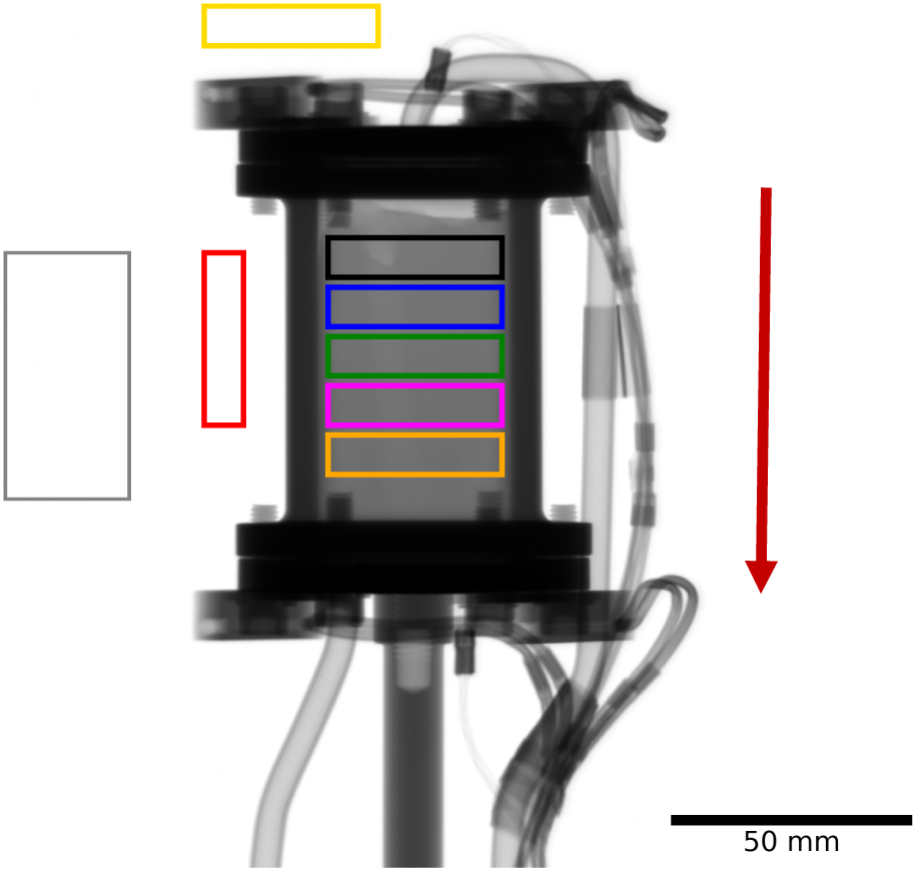
A neutron radiograph of the reactor plus catalyst charge. The colored boxes define the regions of interest (see text). Collectively, the gray values of the different boxes indicate the spatial distribution of hydrogenous-species concentration across the length of the bed. The red arrow denotes the direction of the flowing gas inside the reactor. The dimensions of the catalyst bed are approximately 40 mm (W) × 65 mm (H) × 13 mm (D).

Once these preprocessing steps had been completed, a further Python script was used to analyze and plot the runs; specifically, to examine the changes in neutron intensity (gray value) of various RoIs as the run progressed. A Savitzky-Golay smoothing algorithm was then applied to the resulting gray value lines to aid in the clarity of the plots. Finally, each interrogated RoI is color-coded to match the line in the gray value plot. These RoIs were chosen to investigate a variety of locations in the catalyst bed, with areas external to the reactor (as signified by red and yellow rectangles) acting as “internal” references.

The primary parameter used to define the characteristics of the trends observed was the % change in gray value intensity (δGV, %) observed on initiation of a perturbation event relative to the normalized preperturbation value (i.e., % change in neutron transmission). This is further discussed in Section 3.1.1.

Lastly, difference frames were generated by subtraction of the reference frame from individual radiographs. These were then labeled with appropriate timestamps for reference with the temporal plots.

A theoretical consideration of the effect of gas flow through an empty cell is considered. Using eq 6 the total drop in neutron transmission caused by 13 mm path length of pure H_2_ gas at standard temperature and pressure is approximately 0.5%. The effect of this path length of He gas is negligible due to the much reduced scattering cross-section for He. The maximum concentration of H_2_ used in this study is ∼ 23% H_2_ in He. Thus, the maximum reduction in neutron transmission of an empty cell caused by gas used in this study is approximately 0.1%, and the majority of measurements use a much lower concentration of H_2_. As 0.1% is essentially the detection limit of IMAT, the effect of the gas itself on the intensity of transmitted neutrons can be considered negligible.

## 3 Results and Discussion

Figure 1 shows a neutron radiograph of the reactor plus catalyst and reveals that, overall, and within the spatial resolution of the measurement, the bed is homogeneous and uniformly packed. This is observed by the approximately constant level of transmitted neutron intensity (i.e., brightness of the image). An exception is a slightly brighter zone toward the top left-hand corner of the bed which extends to a much lesser extent down the length of the reactor. This small region could arise from the presence of silica wool used in the packing of the bed or, alternatively, it may represent an area of slightly less dense packing of the bed. Figure 1 also includes a series of colored rectangles to signify regions of interest (RoI) across the length of the bed. The colors of the RoIs are retained throughout this article to define spatially resolved neutron grayscale values shown in the temporal plots presented in Sections 3.1–3.3. In this way, the averaged gray value (normalized neutron transmission intensity) within the black box indicates the degree of neutron absorption at the entrance of the reactor (top of the catalyst bed), while the averaged gray value within the orange box signifies the degree of neutron absorption at the exit of the reactor (bottom of the catalyst bed). Measured neutron transmission within the red and yellow boxes external to the reactor are used as control regions and the gray region is used for purposes of normalization to neutron beam intensity fluctuations as described in Section 2. The length of the catalyst bed is approximately 62 mm from top to bottom. Taking the midposition of each box therefore describes a progression along the catalyst bed from the gas entry to gas exit points. Table 1 shows the extent of progression across the length of the catalyst bed that each RoI represents.

**Table 1:** Key Features of the Curves Depicted in Figure 2b, Describing the Kinetics of Hydrogen Adsorption

RoI color	Midpoint length (mm)	δGV_max_ (%) ± 0.1%	Δt_max_ (min) ± 0.5 min	Δt_lag_ (min) ± 0.5 min	t(δGV) (% min^–1^) ± 0.02% min^–1^
Black	8	2.8	11.5	-	0.43
Blue	18	2.7	16.0	3.5	0.34
Green	28	2.7	20.5	3.5	0.26
Magenta	38	2.9	28.0	3.5	0.18
Orange	48	3.1	35.0	3.5	0.15

### 3.1 The Interaction of Hydrogen with the As-Received Catalyst

#### 3.1.1 Ambient Temperature Measurements

These initial measurements are intended to see how the as-received (i.e., unactivated) catalyst responds to being exposed to hydrogen in a helium carrier flow, thereby providing spatial and temporal information on various parts of the catalyst reduction stage. Figure 2a and b respectively present the mass spectrometer output and spatially distinct neutron transmission levels as a function of time-on-stream (T-o-S). Throughout all the measurements undertaken, the catalyst is continually flushed by a flow of helium at a flow rate of 100 mL min^–1^. For Figure 2, the catalyst is at room temperature (293 K). Hydrogen at a flow rate of 10 mL min^–1^ is introduced alongside the He carrier gas at t ∼ 5 min, as indicated by the dashed red line.

**Figure 2:**
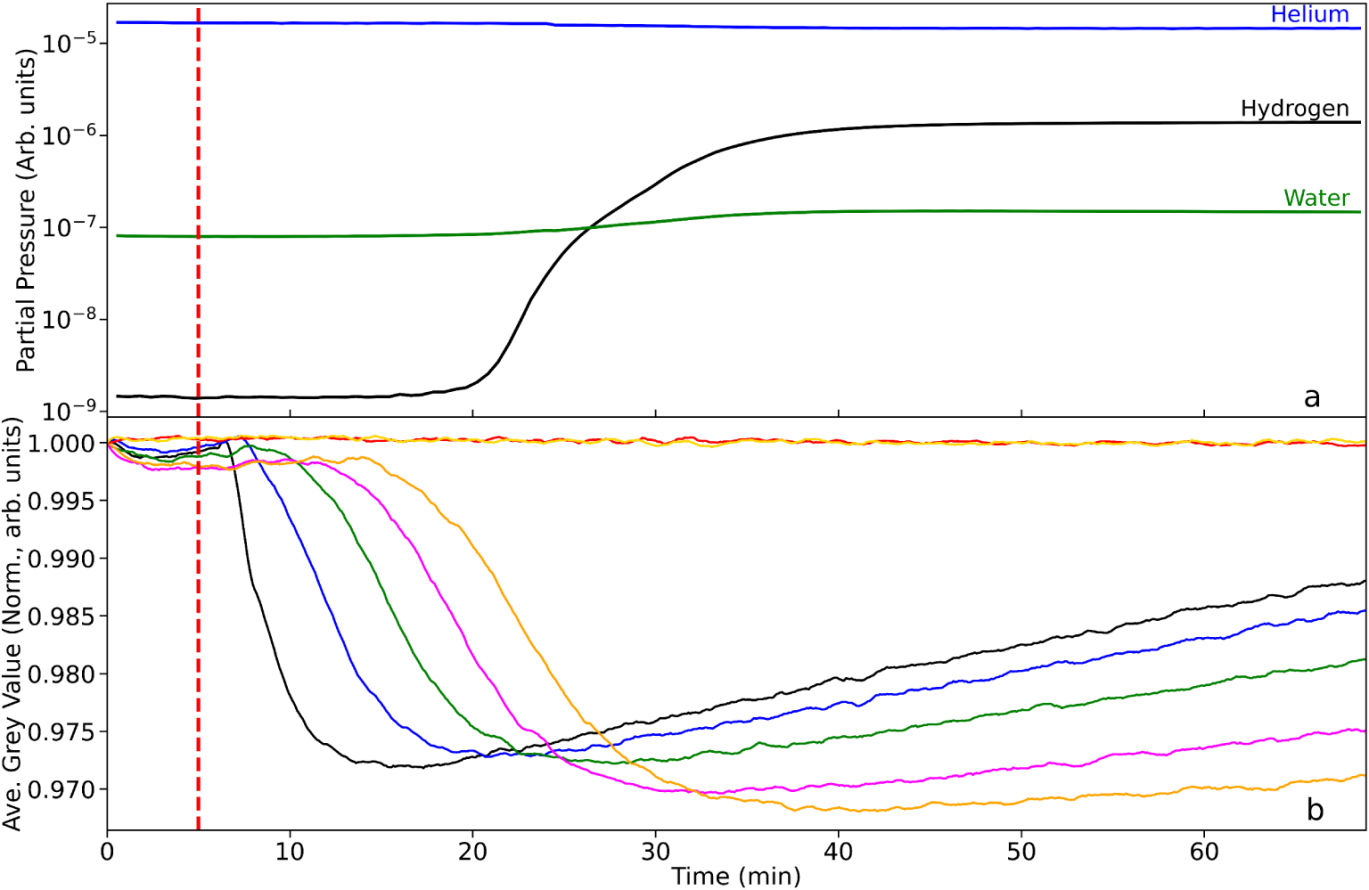
(a) Mass spectrometric profile for the exit gas as a function of T-o-S and under constant He flow (100 mL min^–1^): the as-received catalyst is at room temperature; hydrogen gas (10 mL min^–1^) is introduced into the helium feedstream at t ∼ 5 min (dashed red line). (b) Corresponding average neutron grayscale values for each RoI. The linkage between the color of the neutron intensity plots and their respective location along the reactor are defined in Figure 1.

Figure 2a shows that the hydrogen is not detected at the rear of the reactor until t ∼ 20 min. Thereafter, over a period of ∼ 20 min the hydrogen concentration progressively increases to a plateau level at t ∼ 40 min. In this way, it takes 15 min for the hydrogen to pass through the whole of the catalyst bed and, thereafter, it requires 20 min for saturation of the Pd-hydrogen adsorption complex to be achieved. The hydrogen signal for T-o-S ≥ 40 min is thought to indicate equilibrium at the solid/gas interface, and signifies the condition,

1 $$\frac{k_{a}}{k_{d}}=K$$

(K is an adsorption coefficient, *k*_a_ is the adsorption rate coefficient and *k*_d_ is the desorption rate coefficient), where the sum of the adsorption rate and the desorption rate is zero.

The profile in Figure 2a is equivalent to a “breakthrough” measurement and indicates the interaction between the hydrogen gas and the as-received catalyst to be a relatively slow process. On reactor charging, the catalyst is thought to be comprised of Pd crystallites primarily present in a 2+ oxidation state (Pd^2+^), with the Pd present as Pd oxide (PdO). The small increase in the water signal seen in Figure 2a at t ∼ 36 min indicates that even at ambient temperature (293 K), a small degree of reduction of the Pd particles and drying of the catalyst is taking place. Collectively, Figure 2a presents the integrated response of the reactor in terms of hydrogen adsorption to the Pd/Al_2_O_3_ catalyst at 293 K.

Figure 2b presents the associated neutron imaging intensities of the spatially distinct zones of the reactor, where the colored lines correspond to the integrated intensity of the 7 RoIs, as presented in Figure 1. Note, the progression from the top of the reactor to the bottom of the reactor is signified by the following color order: black > blue > green > magenta > orange. Moreover, the two plots colored red and yellow correspond to control areas outside of the reactor and, therefore, should not display any chemistry and associated change in neutron intensity. Validating that assumption, the red and yellow plots in Figure 2b show no change in gray value over the full 75 min duration of the scan period. This contrasts dramatically for the plots conveying information on hydrogen concentration within the reactor. All five line plots possess a similar profile, namely a steep decrease toward a minimum gray value followed by a more gradual increase in gray value. However, there is a clear phase shift between regions. In order to define these characteristics, δGV is used to describe the maximum decrease in gray value, Δ*t*_max_ represents the time from hydrogen injection that the δG*V*_max_ corresponds to, Δt_lag_ represents the time lag between the 50% δGV value for a RoI and the 50% δGV value of the previous RoI and t­(δGV) represents how quickly the GV for each RoI dropped from its maximum value to δG*V*_max_, i.e., it is a rate of neutron attenuation that corresponds to hydrogen adsorption. Table 1 presents the δG*V*_max_, Δ*t*_max_ and Δt_lag_ values for Figure 2b. A visual indication of what each column represents can be seen in Figure S2.

The black box, which is closest to the gas entry point of the reactor, sees a deflection in the neutron gray value first. The decrease in gray value on hydrogen addition is significant (δG*V*_max_ = 2.8% drop in neutron intensity). The decrease is associated with an increase in hydrogen concentration in this RoI, where the increased concentration leads to greater neutron attenuation, which manifests itself as a decrease in gray value. Figure 2b shows the onset of the decrease to occur approximately 2 min after the introduction of the hydrogen, indicating the hydrogen adsorption in the first RoI to be a reasonably rapid process. The steep negative slope of the black box gray value as a function of T-o-S indicates the accumulation of hydrogen within the RoI to be rapid. The minimum in the profile of the gray value for the black box corresponds to a Δ*t*_max_ value of 11.5 min, and thereafter there is a progressive and seemingly linear increase in the gray value, so that at the scan end of 70 min, the gray value represents only ∼40% of δG*V*_max_. This progressive decrease in neutron attenuation signifies a progressive reduction in hydrogen concentration in the RoI. This could occur from hydrogen desorption or, alternatively, it could indicate a more complex hydrogen transformation process as well as some continued drying of the water formed from any reduction and from the as-received catalyst.

The blue cell represents a progression down the reactor in the direction of hydrogen flow. Although the form of the blue curve mirrors that of the black curve, Figure 2b and Table 1 show the blue curve to exhibit a significant time lag (Δt_lag_) with respect to the onset of hydrogen addition. This effective phase shift in the concentration profile is attributed to physicochemical processes that are delaying the transport of hydrogen gas as it passes down the length of the bed. Table 1 shows the Δt_lag_ values to be broadly consistent for all RoIs (Δt_lag_ = 3.5 (±0.5) min). This is thought to represent the gross transport and diffusion resistance encountered by hydrogen across the bed. However, one acknowledges that trends evident in Figure 2b show that hydrogen transport along the bed is not solely down to hydrogen diffusion through the solid matrix coupled with hydrogen adsorption. Moreover, as seen with the black line plot, upon reaching its Δ*t*_max_, the blue line similarly exhibits a linear increase in gray value, achieving 50% of δG*V*_max_ at the scan-end. With respect to the black line plot, this represents a decrease in reduction of δG*V*_max_, signifying a retardation in the process responsible for the up-tick. This trend continues as one progresses down the bed, with the orange line plot exhibiting a grayscale value at scan end comparable to its δG*V*_max_. Evidently, the process, or processes, responsible for the up-tick are much slower at the rear of the bed than is observed at the front of the bed.

In order to identify the contributors to the impediment of hydrogen transport through the Pd/Al_2_O_3_ catalyst at ambient temperature, we need to better understand the origins of the up-tick in the hydrogen concentration curves (Figure 2b), which signifies a reduction in hydrogen concentration for T-o-S values > Δ*t*_max_. Figure 2a is informative here. Examination of the water signal as a function of T-o-S shows that the H_2_O partial pressure before hydrogen is introduced to the reactor is unchanged up to approximately t = 35 min. At which point there is a modest step change to increased partial pressure that, thereafter, remains approximately constant to scan-end. As noted above, the presence of water is attributed to a combination of catalyst drying and hydrogen-induced reduction of PdO. Interestingly, with reference to Table 1, t = 35 min roughly corresponds to the Δ*t*_max_ value for the orange curve. Thus, water production is only detected in the exit gas once the bottom zone of the catalyst bed has completed the hydrogen adsorption stage. In this way, Figure 2 is showing the totality of processes encountered as hydrogen is passed over an extended bed of Pd/Al_2_O_3_ catalyst. The first process is physical in nature and is the diffusion/transport of H_2(g)_ through the porous network of the alumina-supported Pd catalyst. This is a relatively fast and physical process that is expected to be a constant throughout the whole bed. Figure 2a and b imply that chemical processes are complicating the transport of hydrogen throughout the catalyst bed. eq 2 defines the hydrogen adsorption process and eq 3 describes the hydrogen reduction process. Importantly, the formation of water is taking hydrogen away from the reactor.

2 $$H_{2\mathrm{(g)}}⇌2H_{\left(\mathrm{ad}\right)}$$

3 $${\mathrm{PdO}}_{\left(s\right)}+2H_{\left(\mathrm{ad}\right)}\to {\mathrm{Pd}}_{\left(s\right)}+H_{2}O_{\left(g\right)}$$

The t­(δGV) column of Table 1 is an indicator for the rate of the adsorption process (eq 2), which ranges from 0.43 to 0.15% min^–1^ on transversing from black to orange boxes, indicating that hydrogen adsorption is fastest at the front of the bed. When considering the increase in gray values for *t* > *t*_max_, it was noted above that hydrogen desorption could be a contributor. However, that process is contained within eq 2 (the reverse reaction within the equilibrium) and is thought not to be responsible for the up-tick seen in Figure 2b. Rather, a combination of drying under a flow of dry He and eq 3 is thought to be responsible for the increase in gray value at T-o-S > *t*_max_. Supported Pd catalysts can be reduced at relatively low temperatures (<100 °C).^17^ So, in combination with simple drying of the as-received catalyst, it is the production of water, taken away from the catalyst in the presence of a continuous purge gas, that reduces the hydrogen content, thereby leading to an increase in neutron transmission as measured by the detector.

On inspection of Figure 2b, two factors indicate that the rate of the reduction process is diminishing along the length of the bed. First, the t­(δGV) column of Table 1 shows the black region to exhibit the steepest slope with the gradient decreasing on increasing bed length. Second, as noted previously, the gray value at scan-end (T-o-S = 75 min) is lower the further down the bed one goes. The reason why the reduction process is slower at the back of the reactor compared to the rate observed at the front is uncertain. However, one possibility could be that the relatively slow hydrogen flow rate (10 mL min^–1^) is being consumed as the hydrogen gas advances along the bed to produce Pd hydride (eq 4). This process consumes the hydrogen and reduces the hydrogen available at the end of the bed to maintain eq 3.

4 $$H_{\left(\mathrm{ad}\right)}+\mathrm{Pd}⇌\mathrm{Pd}-H$$

Additionally, as the catalyst is dried by the flow of gas, this gas is collecting water initially from the regions near the top of the reactor first. This wet gas is then retarding the drying of the catalyst in lower regions of the reactor, causing a reduced rate of drying in these lower regions and resulting in less of an up-tick in the plots in Figure 2b.

Figure 3 presents a sequence of neutron “difference” radiographs recorded over the period of 0 – 70 min as a function of T-o-S, with increasing darkness signifying increasing neutron attenuation, i.e., greater δGV. 0.0 min represents a reference state with uniformity evident throughout the bed. At t= 7.2 min, and all subsequent frames there is a thin light patch at the top of the catalyst bed caused by a small amount of bed compaction, i.e., the catalyst is drawn down slightly leaving a less dense region of increased neutron transmission where the catalyst is now missing. At t = 7.2 min there is also a darkening at the top of the bed. This corresponds to a combination of the compacted catalyst bed and the hydrogen adsorption phase within the black ROI (Figure 2b). At a time of 15 min, the data are characterized by reduced neutron transmission (darkness) in the top half of the reactor, signifying high hydrogen concentrations in this region. There is reduction in this darkness from approximately halfway down the reactor, with the bottom third displaying the same low level of gray value seen in the reference (t = 0 min). Fifteen min is thought to broadly correspond to a response up to the *t*_max_ value as presented for the green plot in Figure 2b. At t = 30 min there is uniform attenuation throughout the whole reactor, showing the catalyst to be saturated with hydrogen at this point. Interestingly, at t = 70 min there is a reversal of the trend displayed in the previous images: the top part of the reactor has diminished grayness (lower hydrogen concentration), whereas the bottom part of the reactor retains gray values comparable to that evident at t = 30 min. These changes in neutron transmission signify that the top part of the reactor is losing hydrogen (via eq 3 and drying), while the bottom part of the reactor is experiencing more of an adsorption phase than a reduction phase. The full video of the frames represented in Figure 3 is available in Supporting Information.

**Figure 3:**
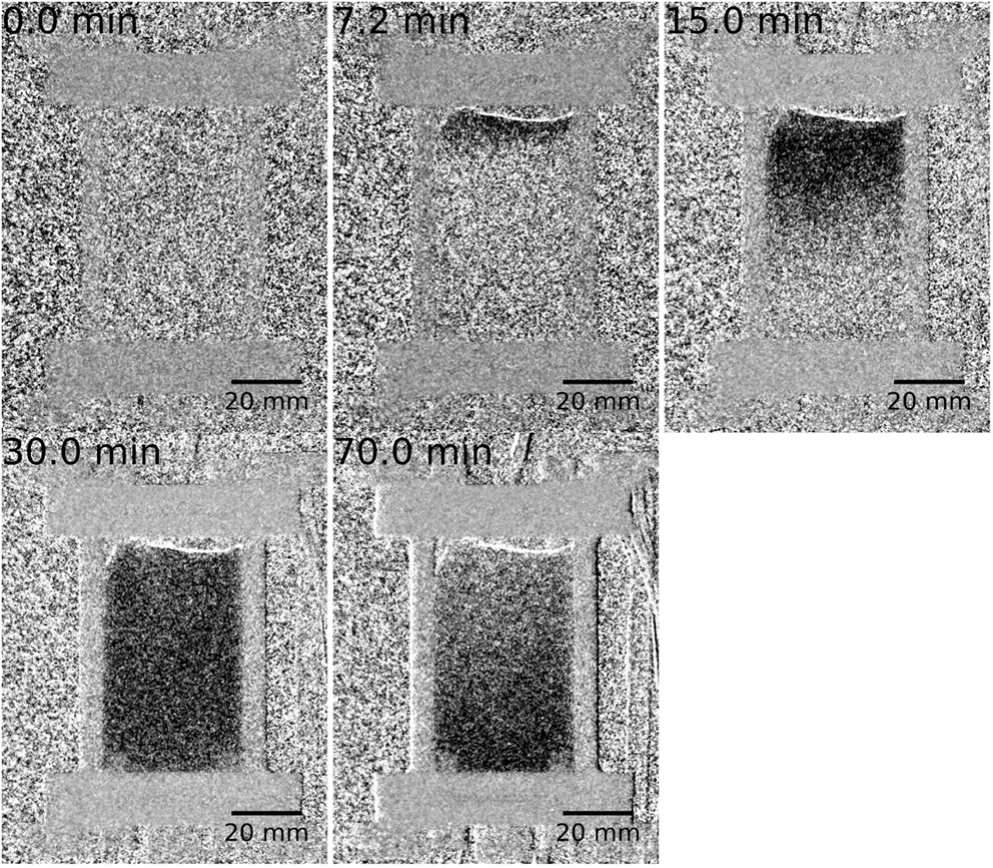
A sequence of neutron “difference” radiographs recorded over the period of 0 – 70 min as a function of T-o-S during which the as-received catalyst is maintained at ambient temperature and hydrogen (10 mL min^–1^) is introduced into the helium feedstream (100 mL min^–1^) at t ∼ 5 min. The image of the reactor at time *t* = 0 min is subtracted from the image at a particular T-o-S: 0, 7.2, 15, 30, and 70 min. Increasing darkness signifies decreasing neutron transmission due to spatially resolved increasing hydrogen content.

Considering profiles presented in Figures 2 and 3, it is clear that under the stated reaction conditions, the adsorption stage precedes the reduction/drying stage and that the former is a faster process than the latter. On acknowledging that catalyst reductions are normally conducted at elevated temperatures,^17^ it is presumed that increasing temperature will lead to an increase in the rate of reaction 3 and, hence, more efficient reductions and faster drying.

#### 3.1.2 Quantification of Hydrogen Absorption

The data presented allow for the quantification of hydrogen absorption via two independent methods: the first using the known flow rate of H_2_ gas and the mass spectrometry data; the second using the neutron attenuation values measured in the radiographs.

Initially, there is no H_2_ in the catalyst or the He feedstream. Flow of H_2_ is then introduced at a known rate, i.e., 10 mL min^–1^. As the mass spec trace for H_2_ remains unchanged upon this introduction of gas to the feed stream, we can correlate this mass spec signal with complete absorption of H_2_ by the catalyst bed, i.e., the absorption rate is 10 mL min^–1^. At late stages of the experiment, where the mass spec signal for H_2_ plateaus, it can be assumed that the catalyst is fully saturated with hydrogen and the signal corresponds to a flow of 10 mL min^–1^ being passed through the reactor without loss, i.e., 0 mL min^–1^ H_2_ is being absorbed. It is, therefore, possible to convert the mass spectrometry signal for H_2_ to units of “H_2_ absorption rate”, retaining units of ml min^–1^ by simply inverting the mass spec trace and assigning the rates of absorption to 10 mL min^–1^ at the start and 0 mL min^–1^ at the end. If the integral of this is taken between the onset of H_2_ absorption, as determined by the onset of neutron transmission decrease in Figure 2b, and the plateau then a total volume of absorbed H_2_ gas can be obtained. These data are plotted in Figure 4.

**Figure 4:**
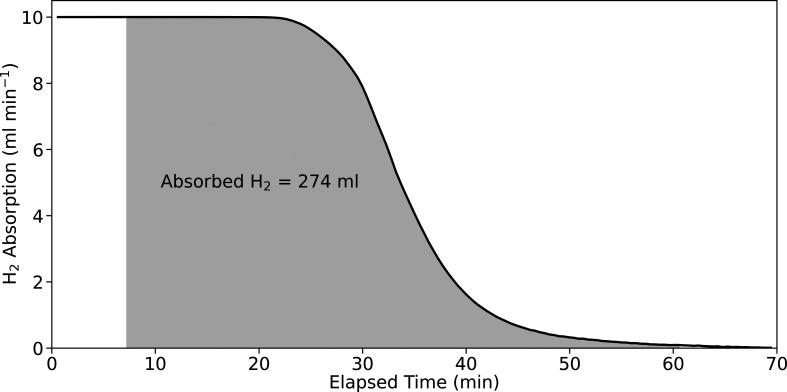
Inverted mass spectrometry data for H_2_ showing the rate of H_2_ absorption by the catalyst as a function of time at 293 K (Figure 2). The gray shaded area corresponds to the limits of the onset of H_2_ absorption and the plateau at H_2_ saturation, and the integrated area is given in units of volume of H_2_ absorbed.

Knowing the cell temperature of 293 K and gas pressure of 1 bar, it is possible to use the ideal gas law to estimate the number of moles of H atoms absorbed over this time period. In this manner, we obtain a value of 23 mmol of H atoms.

The second method utilizes the Beer–Lambert law for neutron attenuation, as given by eq 5:

5 $$\frac{I}{I_{0}}=\exp\left(-n\sigma_{T}t\right)$$

where *I* and *I*_0_ are the full and attenuated transmission intensities respectively, *n* is the number density of atoms, σ_*T*_ is the total neutron scattering cross section for the atoms, and *t* is the path length of the sample. This can be rearranged to obtain *n*,

6 $$n=\frac{-\ln\left(\frac{I}{I_{0}}\right)}{\sigma_{T}t}$$

For 3 Å neutrons, the value of σ_*T*_ for H is 82.574,^18^ *t* is 13 mm (see Section 2.1), and the relative neutron attenuation fraction can be directly read from the gray values in Figure 2b. Observing that the minimum value for all RoIs is approximately the same and assigning it a value of 0.972 for *I/I*_0_, we can use the volume of catalyst to obtain a value of 29 mmol of H atoms absorbed throughout the entire catalyst bed.

These two values, 23 and 29 mmol, are clearly not identical. A number of factors increase the uncertainty in these calculations, such as dead volume in the capillaries and reactor, and the aforementioned drying of the catalyst leading to differences in neutron attenuation. However, the results are comparable and determined independently of each other, affording confidence that they are representative of the approximate amount of hydrogen absorbed. Such uncertainties can and will be addressed in future experiments.

Importantly, for 29.2 g of a 5 wt % Pd catalyst, we have a total of approximately 1.46 g (13.7 mmol) of Pd metal. Pd is known to form the hydride PdH_*x*_ at room temperature,^19^ with an approximate value of x = 0.7 for 1 bar H_2_ at room temperature.^20^ Formation of the hydride could then account for approximately 10 mmol of H atoms. As this is a significantly lower value of H atoms than appears to be adsorbed in the sample, it is clear that the majority of adsorbed hydrogen is being used to reduce the PdO species, as in eq 3. This H_2_O initially remains in the catalyst bed before being removed by the continued flow of He/H_2_ and additional heating, described in the following section.

This method of quantification of absorbed species through both mass spectrometric and neutron transmission routes is an example of the rich level of information that can be obtained with the *operando* variant of neutron imaging.

#### 3.1.3 Catalyst Activation, Hydrogen Adsorption at 100 °C

Building on the outcomes realized in the preceding section, this section considers how increasing temperature affects the Pd/alumina/hydrogen/water interactions across the length of the bed. The experimental run follows on directly from that presented in Section 3.1.1. Initially, the catalyst is at room temperature (293 K) with a continuous feed of He (100 mL min^–1^) and H_2_ (10 mL min^–1^) passing over it. At t = 15 min, the temperature set point is switched to 373 K. Mass spectrometric (Figure 5a) and neutron imaging scans (Figure 5b) were accumulated during the temperature ramp that took 45 min to achieve the target temperature. Toward the end of the run, at t = 140 min, the hydrogen flow rate was increased to 30 mL min^–1^.

**Figure 5:**
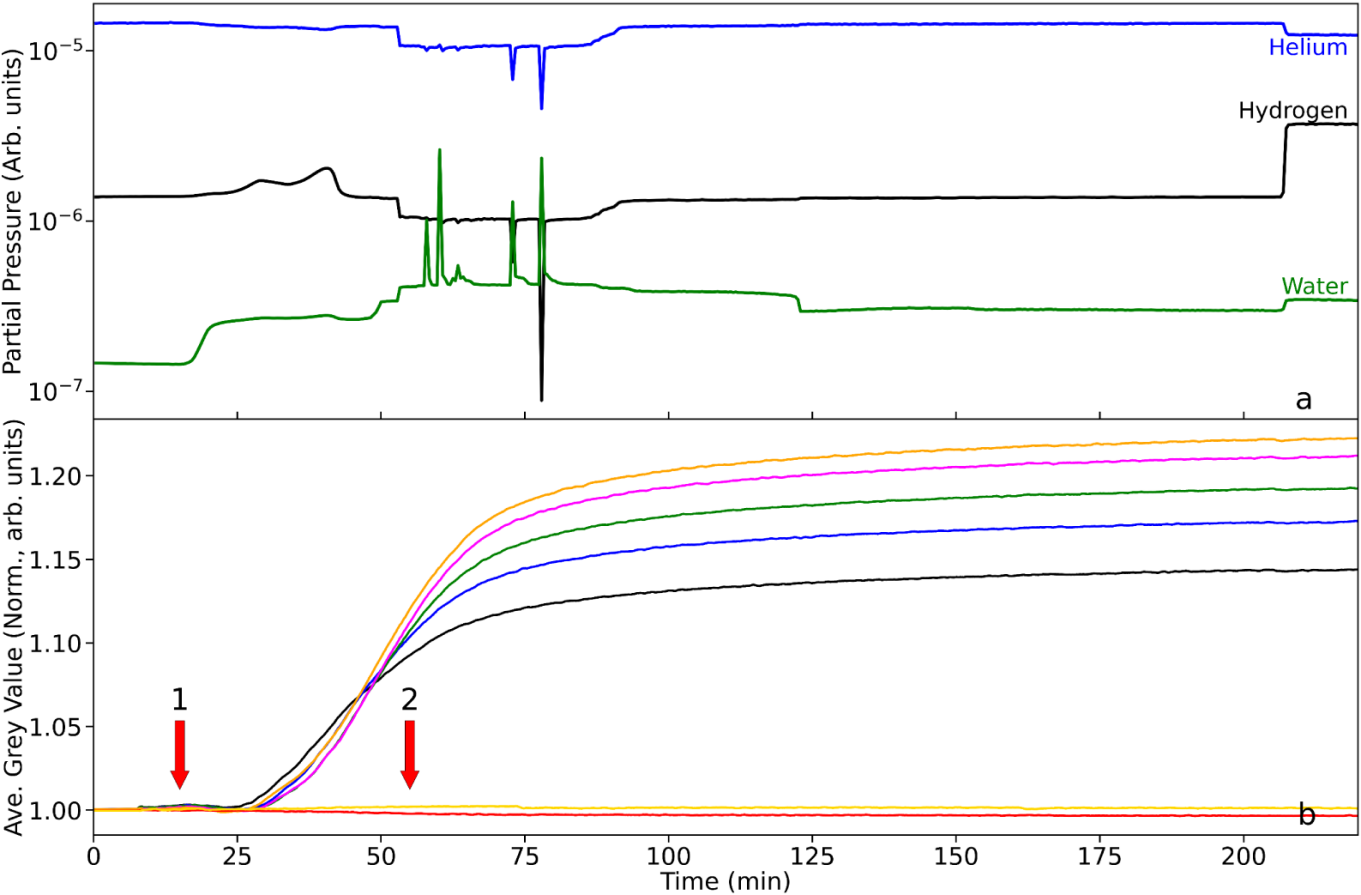
(a) Mass spectrometric profile for the exit gas as a function of T-o-S during a period when the catalyst is warmed from ambient temperature to 100 °C in the presence of a He/H_2_ cofeed (100 and 10 mL min^–1^ respectively) up to *t* = 190 min. At *t* = 200 min, the hydrogen flow rate is increased to 30 mL min^–1^. (b) Corresponding integrated neutron greyscale values. The linkage between the color of the neutron intensity plots and their respective location along the reactor are defined in Figure 1. The arrows represent the times at which the temperature increase begins (1) and the temperature reaches equilibrium at 100 °C (2).

With the occasional spike, Figure 5a shows that increasing the temperature causes fluctuations in the hydrogen consumption, with induced changes in the water signal also evident. In Figure 5b, the temperature increase causes more systematic changes in the gray values of the 5 reactor RoIs but, most notable, is the dramatic increase in neutron transmission (increased gray values). A video of the neutron transmission differences denoted in Figure 5b is available in Supporting Information.

As expected, the red and yellow reference (nonreactor) plots confirm the stability and reliability of the neutron imaging setup, with no change in intensity observed throughout the full scan period. In marked contrast, all five reactor RoIs show an abrupt increase in gray value on commencement of heating. Within approximately 35 min the rate of change in gray value diminishes significantly, and a plateau region is achieved, which is typified by very gradual increases in gray value intensity for increasing T-o-S. In addition, Figure 5b is characterized by a marked gradation of the plateau values that reflects catalyst bed position, with the top of the bed (black plot) returning the lowest gray value (1.14 au) and the RoI at the bottom of the bed (orange plot) the highest (1.24 au). It is important to note that the magnitude of these relative gray value increases (+14–24%) exceed what we usually expect for conventional hydrogen adsorption/desorption processes.

The positive sign of the change in gray value indicates increased neutron transmission that has occurred due to a loss of hydrogen/hydrogenous species from the sample under investigation. This phenomenon is attributed to the temperature increasing the kinetics of the reduction process (eq 3), where water is being formed and then driven out of the reactor by the purge gas. The increase in temperature will increase the rate coefficient for eq 3 and it will also aid the volatilization of the water molecules, thereby ensuring they remain in the gas phase for transportation out of the reactor. The observation in radiographs of pulses of condensed water being observed in the unheated exit pipe of the reactor (Figures S3 and S4) supports this hypothesis. Moreover, this sporadic buildup of water in the exit line that displays condensation/evaporation activity loosely correlates with spikes observed in the mass spectrometer traces. Additionally, it is noted that the increase in neutron transmission for all reactor RoIs is significantly greater than the initial decreases discussed in Section 3.1.1, attributed to hydrogen absorption. Thus, it can be concluded that the greatest effect in this data set is caused by drying of the Pd/Al_2_O_3_ catalyst, resulting in greater neutron transmission than was seen for the as-received sample maintained within a He flow at 293 K.

Indeed, noting that the average increase in neutron transmission across the catalyst bed during this drying process (Figure 5) was of the order of 20%, it is possible to use the total neutron scattering cross section for H_2_O and eq 6 to estimate the amount of water being removed from the system after H_2_ absorption. Doing this results in approximately 45 mmol of water being removed. To demonstrate the additional information that can be obtained from these data we can use this information to quantify the water concentration in the catalyst prior to the start of the experiment. Assuming that a hydride was formed with stoichiometry of PdH_0.7_ as discussed in Section 3.1.1, and this consumed 10 mmol of H atoms, around 13–19 mmol of H atoms remains unaccounted for. Halving this value for the number of H atoms per H_2_O molecule, we can show that approximately 6.5–9.5 mmol of the removed water were formed by oxidation of PdO. This means that approximately half to two-thirds of the Pd in the starting catalyst was in the oxide form. It also shows that between 35.5 and 38.5 mmol of water was removed that cannot be accounted for by reduction of PdO. This means that the catalyst bed contained approximately 640–690 mg of water, i.e., ∼2 wt % of the starting mass of catalyst.

At t = 200 min the hydrogen flow rate was increased from 10 to 30 mL min^–1^, which Figure 5a shows as a step change increase in H_2_ partial pressure that corresponds to a marginal increase in water emission from the reactor. Against this change in the exit gas composition, Figure 5b shows no change to be detectable in the reactor gray values. This is thought to signify that hydrogen demanding processes such as adsorption and reduction are effectively complete after this sample history (sustained hydrogen flow at ambient and elevated temperatures), so that extra hydrogen creates little change to the reactor/catalyst combination. That said, increasing hydrogen exposures could well lead to greater hydride formation (via eq 4) but exploration of that concept is not readily achievable by neutron imaging alone. The inclusion of diffraction and spectroscopic techniques would be useful in this regard.

Figure 5b shows the final gray values to correlate with reactor bed length. Specifically, the final gray values systematically increase on going from top to bottom of the reactor. Indeed, the actual magnitude of the change in final gray value (δGV) is thought to be a contributor to the sensitivity of this measurement to indicate compositional changes along the length of the bed. The return of the water trace in Figure 5a to baseline values after T-o-S ∼ 110 min shows the reduction and drying processes to be complete from then on. There are two potential explanations for the differences seen through the length of the catalyst bed. First, all gray values are normalized to the start of the individual data set. As discussed in Section 3.1.1, the upper most regions of the catalyst bed (black and blue) already showed an up-tick in neutron transmission toward the end of the data shown in Figure 2b. This is not further accounted for in Figure 5, and thus these regions have already undergone some increase in neutron transmission and so the overall relative change in Figure 5b is likely to be less for these RoIs than for those lower in the catalyst bed. Second, it is suspected that the catalyst packing density is not uniform throughout the reactor, with the regions near the top having a lower density than those at the bottom, as one might expect from a simply packed powder held under gravity. This means there may be a greater absolute quantity of catalyst in the RoIs at the bottom of the reactor than at the top, and thus a greater quantity of water is available for drying leading to a larger relative increase in neutron transmission.

### 3.2 Catalytic Activity: Ethene Hydrogenation as a Function of Hydrogen Concentration

Section 3.1 considered the case for hydrogen interactions with the catalyst under conditions of low (293 K) and high (373 K) temperature. This section builds on that platform to consider how neutron imaging can be applied to probe the reactor/catalyst combination during periods of catalytic turnover. Ethene hydrogenation is selected as a representative test reaction.^21^ For all the measurements presented here, the reaction will be performed under isothermal reactor conditions (333 K) but where the relative hydrogen concentration (v/v) will be varied between (i) a large hydrogen-excess regime (hydrogen:ethene = 10:1), (ii) a hydrogen-rich regime (hydrogen:ethene = 5:1), (iii) a hydrogen-lean regime (hydrogen:ethene = 1.7:1) and (iv) conditions where the ethene flow rate is maintained but the hydrogen flow is stopped.

#### 3.2.1 Hydrogen:ethene = 10:1 (V/V)

Following the run presented in Section 3.1.3 (Figure 5), the catalyst temperature was reduced to 333 K, maintaining the helium and hydrogen flows of 100 and 30 mL min^–1^ respectively. At t = 10 min ethene was added to the feedstream at a flow rate of 3 mL min^–1^. With respect to eq 7, the hydrogen:ethene flow rate of 10:1 v/v represents a hydrogen-excess regime.

7 $$C_{2}H_{4}+H_{2}\overset{\mathrm{cat}}{\to }C_{2}H_{6}$$

Figure 6a presents the mass spectrometer profile for the 180 min duration of this run. Ethene intensity is represented by mass 28 amu (magenta) that increases sharply at the ethene injection point. Simultaneously, a decrease in hydrogen concentration is observed (black line), as well as a large increase in ethane (30 amu, orange trace). Following that transitional stage, all the masses outlined above remained broadly at a constant intensity, signifying continuous catalytic turnover of the ethene to form ethane (eq 7). It is important to note that the parent ion fragment for ethane is not 30 amu, but in fact 28 amu. This explains the higher signal for the magenta “ethene” trace in Figure 6a versus the yellow trace for “ethane”. Ethane being produced by the hydrogenation reaction will still afford a high signal in the magenta trace corresponding to 28 amu, however the ratio of the magenta and yellow traces can be observed to identify a change in reaction products, as the yellow trace corresponding to 30 amu is unique to the presence of ethane.

**Figure 6:**
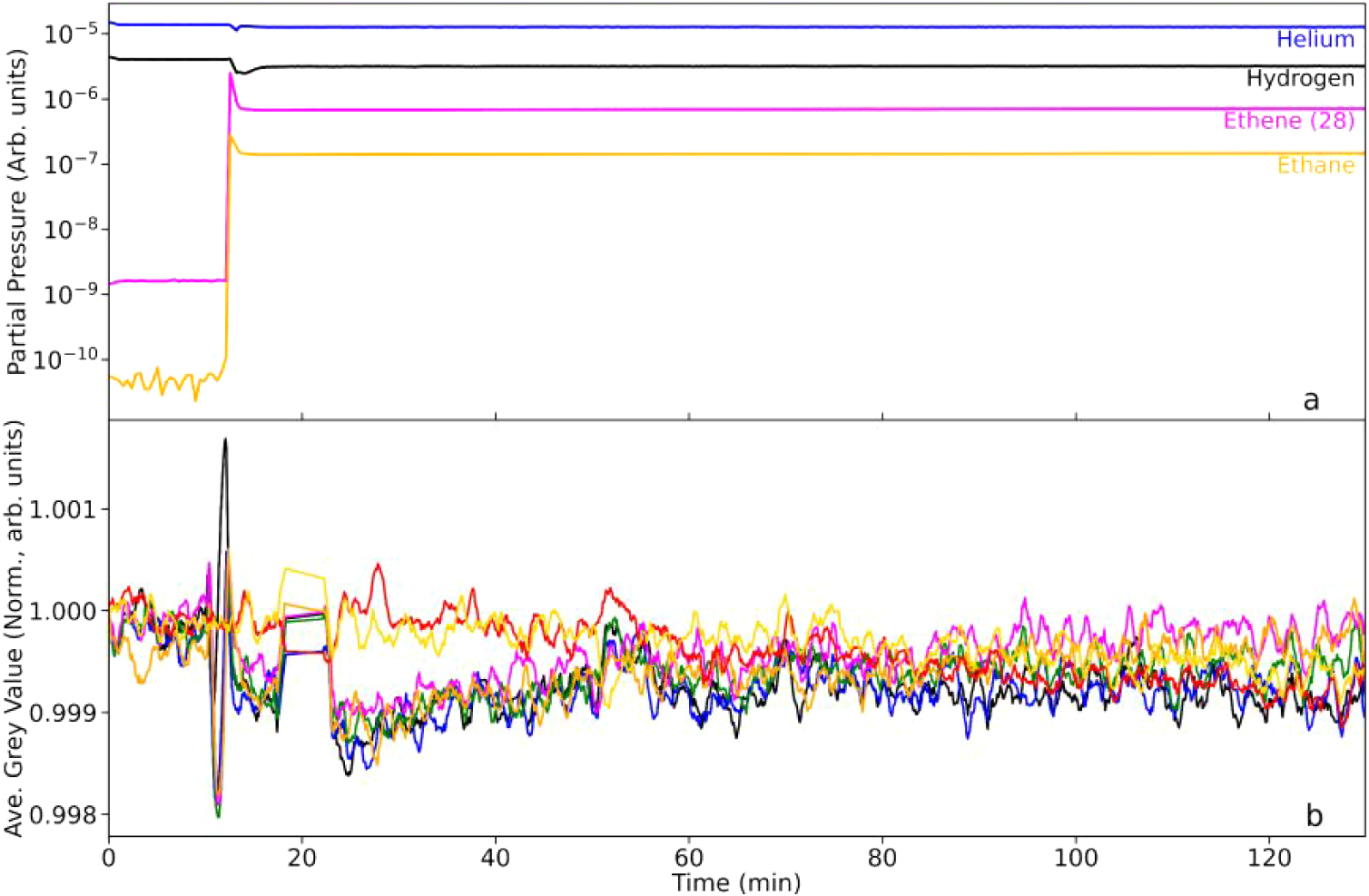
(a) Mass spectrometric profile for the exit gas as a function of T-o-S during a period when the catalyst is maintained at 333 K and with imaging commencing in the presence of a He/H_2_ cofeed (He = 100 mL min^–1^, H_2_ = 30 mL min^–1^). At *t* = 10 min the feedstream is supplemented by the addition of ethene at a flow rate of 3 mL min^–1^, representing a hydrogen:ethene ratio of 10:1 (v/v). (b) Corresponding integrated neutron grayscale values. The linkage between the color of the neutron intensity plots and their respective location along the reactor is defined in Figure 1.

Figure 6b presents the associated neutron imaging profiles for the 5 reactor regions (RoIs) plus the 2 reference volumes (Figure 1). Despite the mass spectrometer profiles presenting logical and expected results, Figure 6b is more difficult to interpret than the neutron transmission plots presented above, with no obvious cohesion between the individual RoIs. Indeed, the maximum decrease in normalized gray value apparent over the scan range is only of the order of 0.05–0.1%, which is close to the sensitivity limit of the neutron instrument. Moreover, the reference regions (red and yellow) exhibit some slight negative drift that occurs within this time period. It is therefore concluded that despite Figure 6a confirming a significant degree of sustained ethene hydrogenation, Figure 6b indicates very little change in hydrogen concentration due to onset of hydrogenation and no obvious spatial distributions as was seen in the activation experiments (Sections 3.1.1-). This is an unexpected outcome that seems to imply there is almost no change in hydrogen concentration within the bed during reaction under these conditions. With respect to eq 7, it is possible that the large hydrogen excess means that there is essentially no accumulation of olefinic species by the catalyst and that the hydrogen consumed in the hydrogenation step is rapidly replenished; the reaction is under steady-state operation.

One aspect that is not shown in Figure 6a is that the partial pressure relating to water is actually seen to decrease very slightly over the course of the run. This was more visible at extended times (Figure S5). In Figure 6b, the return of the gray values to their starting level for the RoIs in the reactor, after the very small initial drop, can therefore be attributed to slow removal of tiny residual quantities of water in the catalyst that were not fully removed by the drying process in Section 3.1.

#### 3.2.2 Hydrogen:ethene = 5:1 (V/V)

This section explores the situation where the hydrogen flow rate has been reduced to 15 mL min^–1^, while maintaining the helium and ethene flow rates at 100 and 3 mL min^–1^ respectively. This corresponds to a hydrogen:ethene ratio of 5:1 (v/v), with the hydrogenation reaction deemed to be occurring in a hydrogen-rich environment, but in far lower excess than for Section 3.2.1. Figure 7 presents (a) the reactor exit pipe mass spectrometer profiles and (b) the spatially defined neutron imaging data as a function of T-o-S. The decrease in hydrogen flow rate is instigated at t = 8 min and is indicated by a red dashed line in Figure 7.

**Figure 7:**
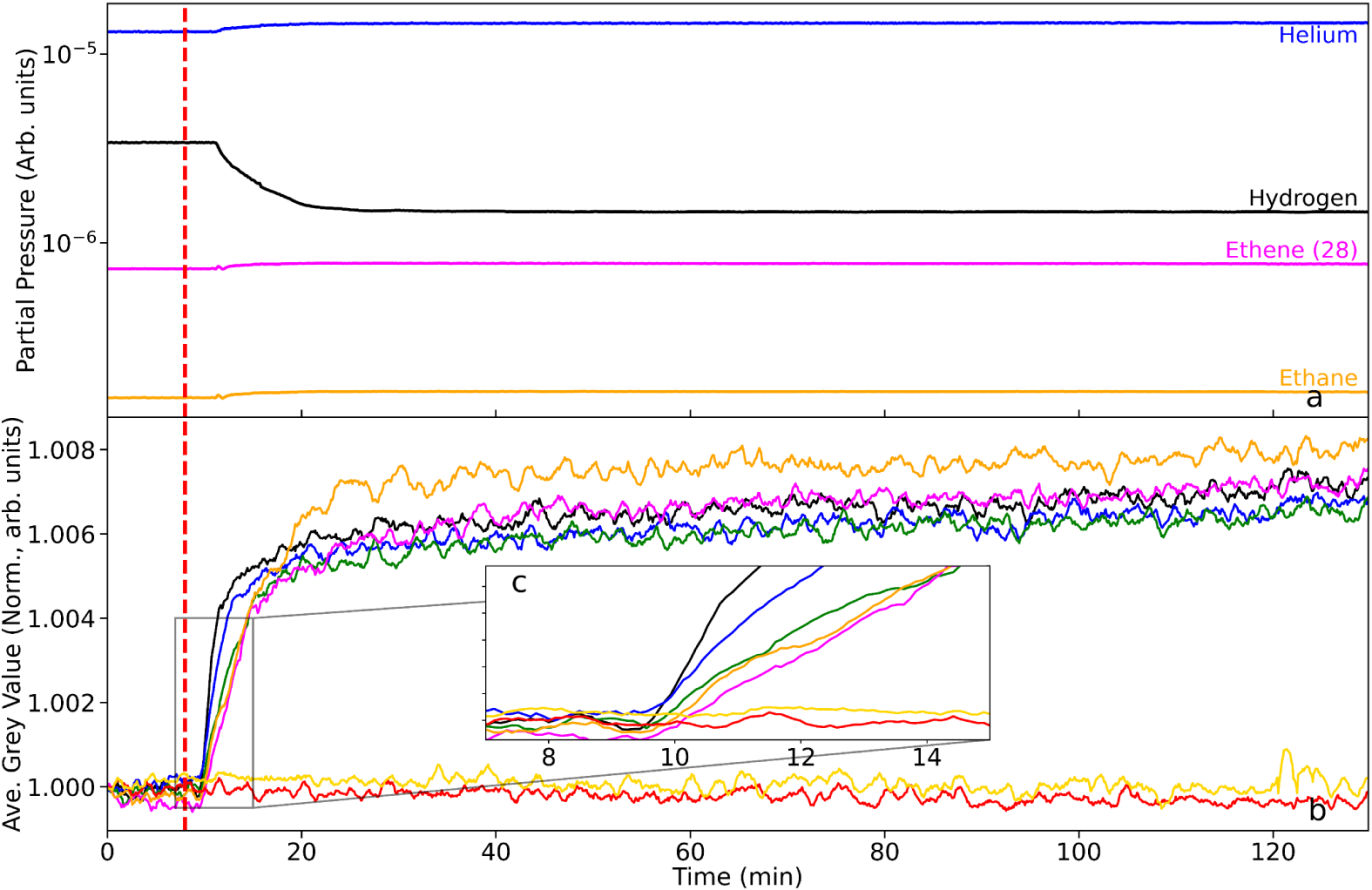
(a) Mass spectrometric profile for the exit gas as a function of T-o-S during a period when the catalyst is maintained at 333 K and with imaging commencing in the presence of a He/H_2_/C_2_H_4_ cofeed (He = 100 mL min^–1^, H_2_ = 30 mL min^–1^, C_2_H_4_ = 3 mL min^–1^). At *t* = 5 min the hydrogen flow rate is reduced to 15 mL min^–1^, representing a hydrogen:ethene ratio of 5:1 (v/v). (b) Corresponding integrated neutron greyscale values. The linkage between the color of the neutron intensity plots and their respective location along the reactor is defined in Figure 1. The dashed red bar denotes the time that the hydrogen flow rate was reduced. (c) Zoomed inset showing the region around the perturbation in greater detail.

The decrease in hydrogen flow rate (black line) is apparent in Figure 7a. At this juncture, there is a small increase in the ethene and ethane signals from that time on, likely due to the change in partial pressure ratio corresponding to a relative increase in ethene and ethane partial pressures, rather than an absolute increase in ethene/ethane quantity. The consistency of the ethene and ethane ratio thereafter establishes that, in the presence of a reduced hydrogen supply, hydrogenation activity is maintained at comparable levels to that observed in the hydrogen-excess regime (Section 3.2.1).

In contrast to the neutron imaging profiles observed in Section 3.2.1 (Figure 6b), which showed no variation upon ethene insertion, Figure 7b shows the reduction of hydrogen flow rate (6:1 →3:1) to exhibit distinct changes in terms of intensity gain (δGV) in the reactor regions stemming from the transition point. Throughout the full scan, the reference signals (red and yellow traces, see Figure 1) are unchanged. The gray value profile for all the reactor RoIs is characterized by a steep increase in gray value over a period of approximately 4 min at the point when the hydrogen flow rate was reduced. This rise is quickly attenuated, with the plots exhibiting a “knee” before plateauing for the remaining acquisition period.

The inset in Figure 7c focuses on the transition point and shows RoIs at the front of the reactor to be the first to register the change in gray value. However, while most of the RoIs reach approximately the same relative change, the orange RoI at the bottom of the reactor, displays a slightly larger relative increase in gray value. The data suggest that the reduction in hydrogen concentration was experienced first at the top of the reactor but, in time, the induced change in hydrogen concentration was slightly greater toward the rear of the reactor. The fact that the δGV increases on dilution of the hydrogen flux means that the neutron transmission has increased, presumably due to a loss of hydrogen from the catalyst. Figure 7b suggests that this was a sudden event that was quickly stabilized, with ethene hydrogenation continuing throughout, as shown in the mass spectrometry. On the assumption that the hydrogenation reaction conforms to a competitive reaction system, the reduction of hydrogen is thought to reduce the relative hydrogen coverage, while the hydrogen supply at the Pd surface remains sufficient so that the rapid hydrogenation reaction is maintained (Figure 7a). This larger relative change for the lower parts of the catalyst bed is also consistent with the previously discussed greater density of catalyst in this region when compared with the upper portion of the reactor.

#### 3.2.3 Hydrogen:ethene = 1.7:1 (V/V)

This section looks at catalytic performance during operation within a more hydrogen-lean regime—hydrogen:ethene = 1.7:1—(helium, hydrogen and ethene flow rates respectively 100, 5, and 3 mL min^–1^). For a period of time, including when the hydrogen flow was reduced, there were technical problems with the neutron instrument meaning that neutron radiography data was not recorded. Neutron data started to be measured again approximately 2 h after the perturbation to the hydrogen flow rate occurred. As such, examination of the neutron data from this point onward without context is not useful, therefore the data plotted in Figure 8 also include the mass spectrometry and neutron transmission included in Figures 6 and 7 to contextualize the data at low hydrogen flow rate.

**Figure 8:**
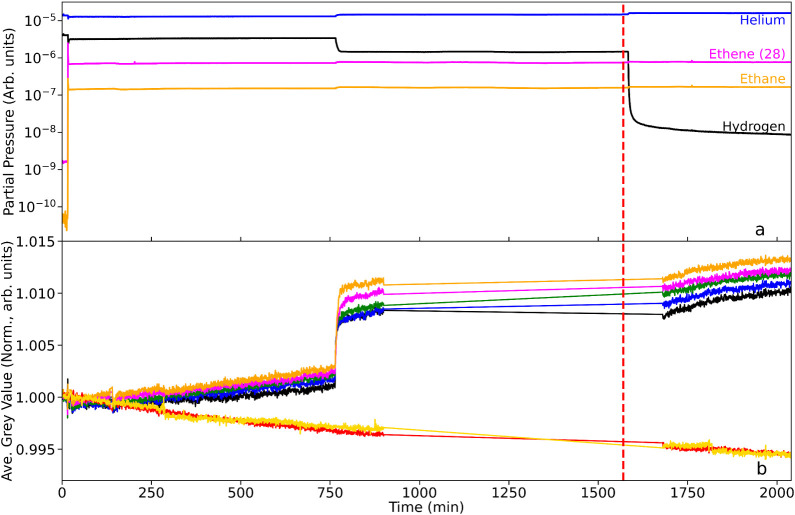
(a) Mass spectrometric profile for the exit gas as a function of T-o-S during the complete ethene hydrogenation experiment with hydrogen:ethene flow rates sequentially 30:0, 30:3, 15:3, 5:3 (ml min^–1^). (b) Corresponding integrated neutron grayscale values. The linkage between the color of the neutron intensity plots and their respective location along the reactor are defined in Figure 1. The neutron data between *t* = 900 and *t* = 1570 min was not recorded due to a fault with the instrument. The dashed red line denotes the approximate point at which the hydrogen:ethene ratio was changed from 5:1 to 1.7:1. The reactor was at 333 K.

Figure 8a shows the complete mass spectrometry data and the sharp drop in the hydrogen signal after the hydrogen flow rate is reduced from 15 to 5 mL min^–1^ is clearly visible at approximately t = 1570 min (dashed red line). Importantly, there is no change in the levels of the ethene and ethane traces, or their ratio, which shows there is still sufficient hydrogen in the system for complete ethene hydrogenation to continue, however, the much reduced flow of hydrogen in the exhaust shown here suggests that very little hydrogen is passing through and exiting the reactor (hydrogen slip).

Figure 8b shows the neutron transmission data recorded throughout the same period of time, with all of the data normalized to the same point used in Section 3.2.1 just prior to the first introduction of ethene, under hydrogen flow at 30 mL min^–1^. By plotting these data all together, the data after t = 1680 min can be better viewed in context. First, despite the missing neutron data, it can be seen that there was no sharp increase in neutron transmission upon reduction of the hydrogen flow and the GV for each RoI in the reactor is approximately the same at t = 1680 min as at t = 900 min. However, there is a slow and steady increase in GV visible at times greater than 1680 min. It is suspected that this is caused by a slow depletion of the hydrogen adsorbed in the catalyst at this lower hydrogen flow rate. It is also noted that the control regions (red and yellow) in Figure 8b show a trend of decreasing GV over time, albeit very slowly at only 0.5% reduction in transmission after more than 33 h. This is likely caused by degradation of the scintillator under constant illumination for such extended periods.

Overall, it would appear that in this system a hydrogen to ethene flow rate of 1.7:1 still leads to complete hydrogenation of ethene to ethane as demonstrated by there being no change in the ratio of signals for 28 amu (magenta) and 30 amu (yellow), however, the data suggest that at a much longer time period than tested in this experiment, the amount of hydrogen adsorbed in the catalyst may reduce enough that this complete hydrogenation is affected. Importantly, then, the ratio of the mass of the catalyst and the overall flow rate of reagent gases has an effect on what can be seen within the time scales of these experiments and must be considered carefully in future.

### 3.3 Hydrogen Starvation Experiments and Reaction Reversibility

#### 3.3.1 Stopping Hydrogen Flow and Maintaining Ethene Flow

After enduring hydrogen-lean conditions (hydrogen:ethene = 1.7:1) for approximately 8 h, the hydrogen flow was stopped while maintaining the ethene flow (helium and ethene flow rates = 100 and 3 mL min^–1^ respectively, Figure 9 dashed red line). This action will stress the reaction system and is intended to perturb hydrogenation activity. The mass spectrometer profiles shown in Figure 9a show the ethane signal to drop abruptly. Over this period the ethene signal increases due to a reduced conversion but the hydrogen profile, somewhat anomalously, exhibits a short-lived dip in intensity that then recovers to preshutdown levels (t = 0–10 min). It is not clear exactly what causes this observation, however one possible explanation follows. Initially, the reduction in hydrogen flow causes a fast drop in hydrogen concentration in the exhaust gas profile. After some time, this reduced concentration of hydrogen in the feed stream may result in stored hydrogen in the catalyst bed (e.g., in the form of PdH), to be released, reestablishing a greater concentration of hydrogen in the exhaust. The decreased ethane signal signifies hydrogenation to have ceased.

**Figure 9:**
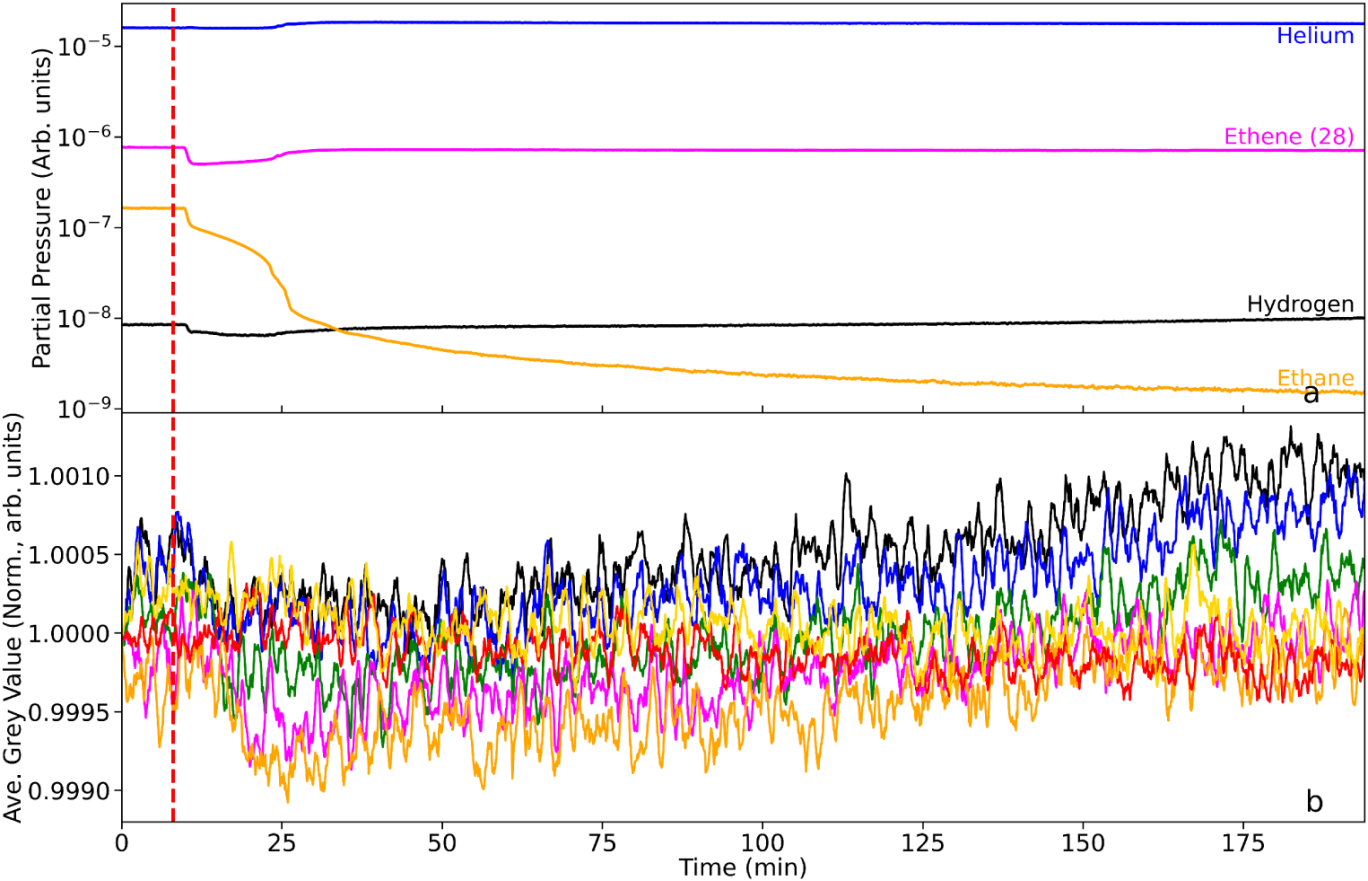
(a) Mass spectrometric profile for the exit gas as a function of T-o-S during a period when the catalyst is maintained at 333 K and with imaging commencing in the presence of a He/H_2_/C_2_H_4_ cofeed (He = 100 mL min^–1^, H_2_ = 5 mL min^–1^, C_2_H_4_ = 3 mL min^–1^), i.e., a hydrogen:ethene ratio of 1.7:1 (v/v). At t ∼ 10 min the hydrogen flow rate is stopped. (b) Corresponding average neutron gray values. The linkage between the color of the neutron intensity plots and their respective location along the reactor is defined in Figure 1. The red dashed line denotes the point at which hydrogen flow ceased.

Figure 9b shows a very weak and gradual increase in gray value for increasing T-o-S, with the front/upper regions of the reactor displaying the greatest rise. As with Section 3.2, this profile is linked to a slow depreciation of hydrogen containing species from the surface. The small change in gray value indicates that the loss of surface hydrogen is minimal, implying that the surface was already well depleted of hydrogen at the start of this data set.

A further investigation of the effect of increasing ethene flow in the absence of hydrogen was undertaken. This can be seen in Supporting Information.

#### 3.3.2 Reversibility Measurements

With an aim of investigating the reversibility of the reaction system, a final flow experiment was undertaken where initially the catalyst was maintained at the high ethene loading (helium/ethene flow rates of 100/12 mL min^–1^). Then at t = 8 min, hydrogen was switched into the feedstream at 20 mL min^–1^, leading to a return to the hydrogen:ethene ratio of 1.7:1, at a higher overall flow rate and concentration than in Section 3.2.3.

Figure 10a is revealing in that, straight away, the ethene signal decreases and the ethane signal dramatically increases. However, there is very noticeable lag in the hydrogen response of the order of 6–7 min. On introduction of hydrogen gas at the entrance of the reactor, a delay of ∼ 5 min occurs before the hydrogen is seen in the exit gas. Meanwhile, during that period efficient hydrogenation has been stimulated.

**Figure 10:**
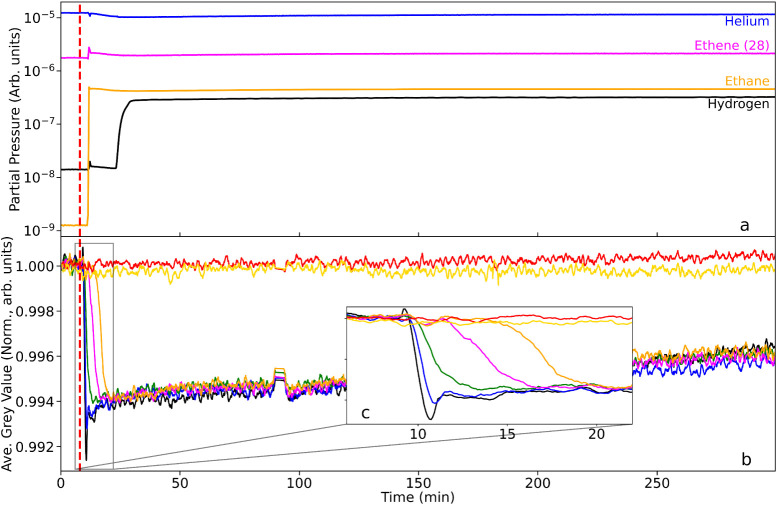
(a) Mass spectrometric profile for the exit gas as a function of T-o-S during a period when the catalyst is maintained at 333 K and with imaging commencing in the presence of a He/C_2_H_4_ cofeed (He = 100 mL min^–1^, C_2_H_4_ = 12 mL min^–1^), i.e., an absence of hydrogen. At t ∼ 8 min the hydrogen flow rate was switched in at a flow rate of 20 mL min^–1^, i.e., a hydrogen:ethene ratio of 1.7:1 (v/v). (b) Corresponding integrated neutron greyscale values. The red dashed line denotes the restart of hydrogen flow. The linkage between the color of the neutron intensity plots and their respective location along the reactor is defined in Figure 1. (c) Zoomed inset showing the region around the perturbation in greater detail.

The reference plots in Figure 10b remain at baseline levels throughout. In contrast, all five reactor segments show a sharp decrease at the hydrogen insertion point. Thereafter the reactor signals show a gradual increase. The initial sharp drop (δG*V*_max_ = 0.6%) is over before the hydrogen signal is registered at the reactor exit. The insert that concentrates on the hydrogen insertion point (Figure 10c) displays a significant dispersion in gray values as a function of position. The decrease in gray value starts first at the top of the reactor (black) then progresses along the bed, with the base of the reactor being the slowest to respond to the hydrogen introduction. This sequence is thought to indicate hydrogen adsorption as it passes through the bed, in an analogous manner to that considered in Section 3.1.1, however to a much-limited degree: δG*V*_max_ = 0.6% cf. 2.5% in Section 3.1.1.

The trends evident in Figure 10b are interpreted as follows. The decrease in gray value signifies an increase in hydrogen adsorption, although the magnitude of the gray value change indicates the extent of this adsorption is modest compared to that observed in the activation measurements (e.g., Figure 2b). It is also noticed how readily the catalyst recovered hydrogenation activity upon the reintroduction of hydrogen (Figure 10a), indicating the hydrogenation process over this catalyst to be reversible under the conditions examined here.

Olefin hydrogenation over supported Pd catalysts is a well investigated phenomenon.^21^ A model of reaction that finds wide acceptance is the concept that hydrogenation does not take place on bare Pd particles but, rather, it takes place over a hydrocarbonaceous overlayer, which forms “pockets” over the metal particles within an initial conditioning process.^22^ These concepts have been expanded upon by Borodziński and coworkers to elaborate on how the hydrocarbonaceous overlayer additionally influences selectivity trends.^23^ Against this background, it is thought that the hydrogen starvation experiments described here have exposed the hydrocarbonaceous overlayer (see Section 3.3.1), which provides a residual signal in the neutron imaging scans. Figure 10b then shows how its activity can be reignited by simply replenishing the hydrogen cofeed. The authors believe that the *operando* neutron imaging experiments outlined here are providing new insight into a well-established topic within heterogeneous catalysis research. The authors additionally note the capability of the technique to interrogate different reactor configurations.

## 4 Conclusions

This work has demonstrated the power of *operando* neutron imaging to investigate aspects of the dynamics of a gas adsorption reaction of a supported Pd catalyst operating in a stainless-steel reactor. The highly penetrating nature of neutrons combined with their sensitivity to hydrogen and hydrogenous materials make neutrons an almost ideal probe for these systems. The kinetics of hydrogen absorption in an as-received catalyst showed two stages: (i) initial hydrogen absorption followed by catalyst reduction and (ii) drying at elevated temperature, with the former proving to be more rapid than the latter. Additionally, a dual approach of neutronic and mass spectrometric analysis allowed quantification of hydrogen absorption to be realized. The uptake of hydrogen gas is spatially and temporally resolved, with distinct transport and diffusion fronts observed.

We have demonstrated the that neutron imaging is well suited for *operando* interrogation of ethene hydrogenation, and that under the studied conditions, the catalyst is able to withstand a wide range of hydrogen:ethene flow rate ratios, including a full stop of the reaction in the absence of hydrogen and the almost immediate reestablishment of catalytic activity when hydrogen flow is restarted. Collectively, these measurements highlight the capability of the neutron imaging technique to assess how hydrogen is being partitioned throughout a catalyst bed during activation and reaction regimes. The fact that this inspection can take place in steel reactors means that reaction engineering issues can be usefully explored via the approach outlined here.

## Supplementary Material







## Notes

A schematic diagram showing the gas flow into, through, and out of the catalytic reactor. Mass spectrometric profile for the exit gas. A series of difference frames taken during the drying of the catalyst (PDF)
Time lapse video of difference frames as H_2_ is introduced to the as-received catalyst (AVI)
Time lapse video of difference frames as the reduced catalyst is dried under He flow (AVI)
