# Supplementary material for: Activation, Steady-State and Passivation Regimes for Ethene Hydrogenation over a Pd/Al 2 O 3 Catalyst: An Operando Neutron Imaging Study
Source: J Phys Chem C Nanomater Interfaces. 2026 Apr 22;130(18):6516–29. doi: 10.1021/acs.jpcc.6c01622 (PMC13158994; doi:10.1021/acs.jpcc.6c01622)
Supplement: Supplementary file 1 [file jp6c01622_si_001.pdf]

Supporting Information for "Activation, Steady-state and Passivation Regimes for Ethene Hydrogenation Over a Pd/Al_2O_3 Catalyst: An Operando Neutron Imaging Study"

Hamish Cavaye[a]*, Christos Ballas[b], Asma Nadia[b], Winfried Kockelmann[a], Stewart F. Parker[a,b], Paul Collier[c], Andrew P.E. York[b,c] and David Lennon[b]

*Hamish.cavaye@stfc.ac.uk
a. ISIS Pulsed Neutron and Muon Source, STFC Rutherford Appleton Laboratory, Chilton, OX11 0QX, UK.
b. School of Chemistry, University of Glasgow, Joseph Black Building, Glasgow G128QQ, UK.
c. Johnson Matthey Technology Centre, Blounts Court, Sonning Common, Reading, RG4 9NH, UK.

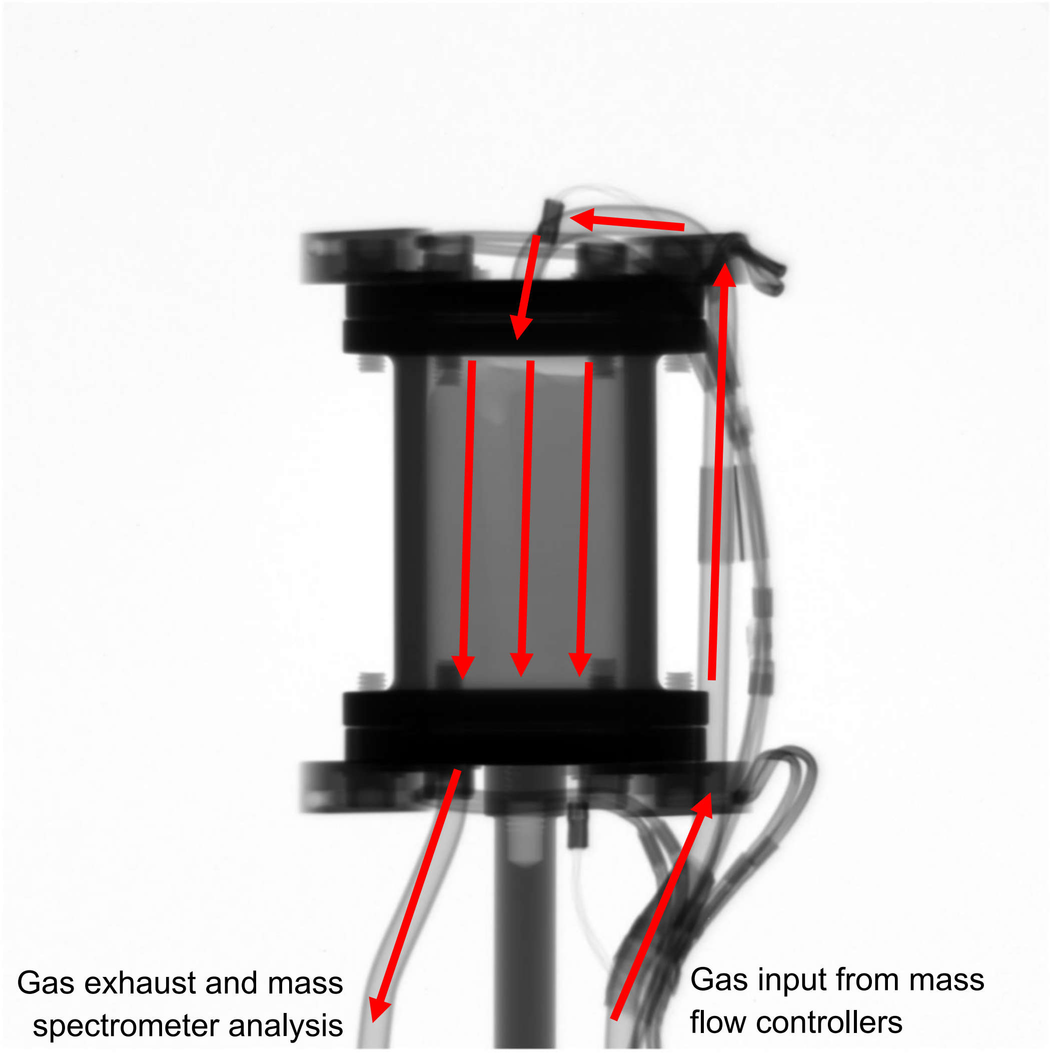

Figure S1. A schematic diagram showing the gas flow into, through, and out of the catalytic reactor.

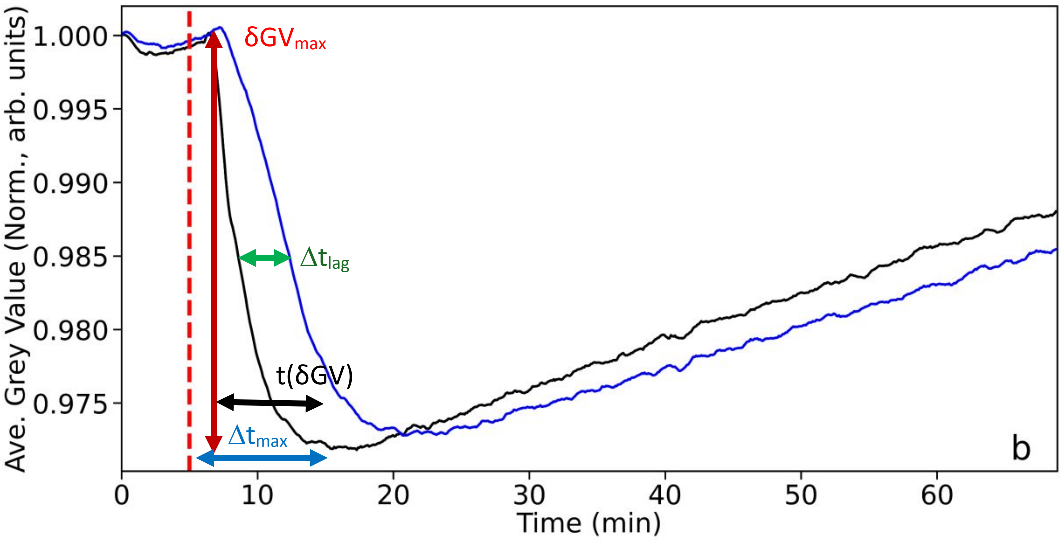

Figure S2. A visual indication of the columns in Table 1 (main manuscript). δGV_{max}, $t(\delta GV)$, Δt_{max} all refer to the black ROI. Δt_{max} relates to the blue ROI as it is 0 by definition for the first/highest ROI.

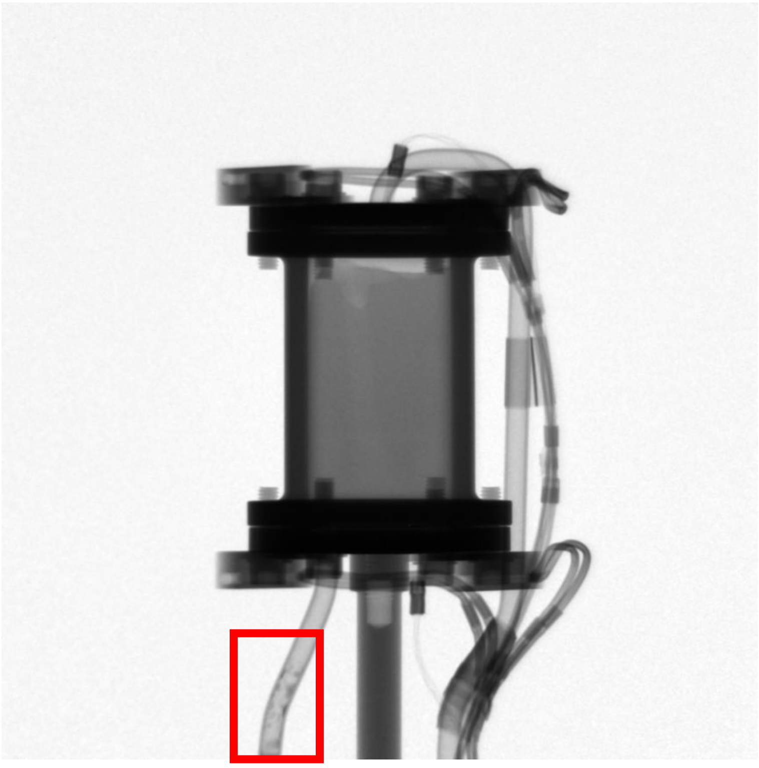

Figure S3. A single calibrated radiograph of the cell heated at 100 °C during hydrogen flow (10 ml min^{-1}) in helium (100 ml min^{-1}) showing water droplets condensing in the unheated exhaust pipe (red rectangle).

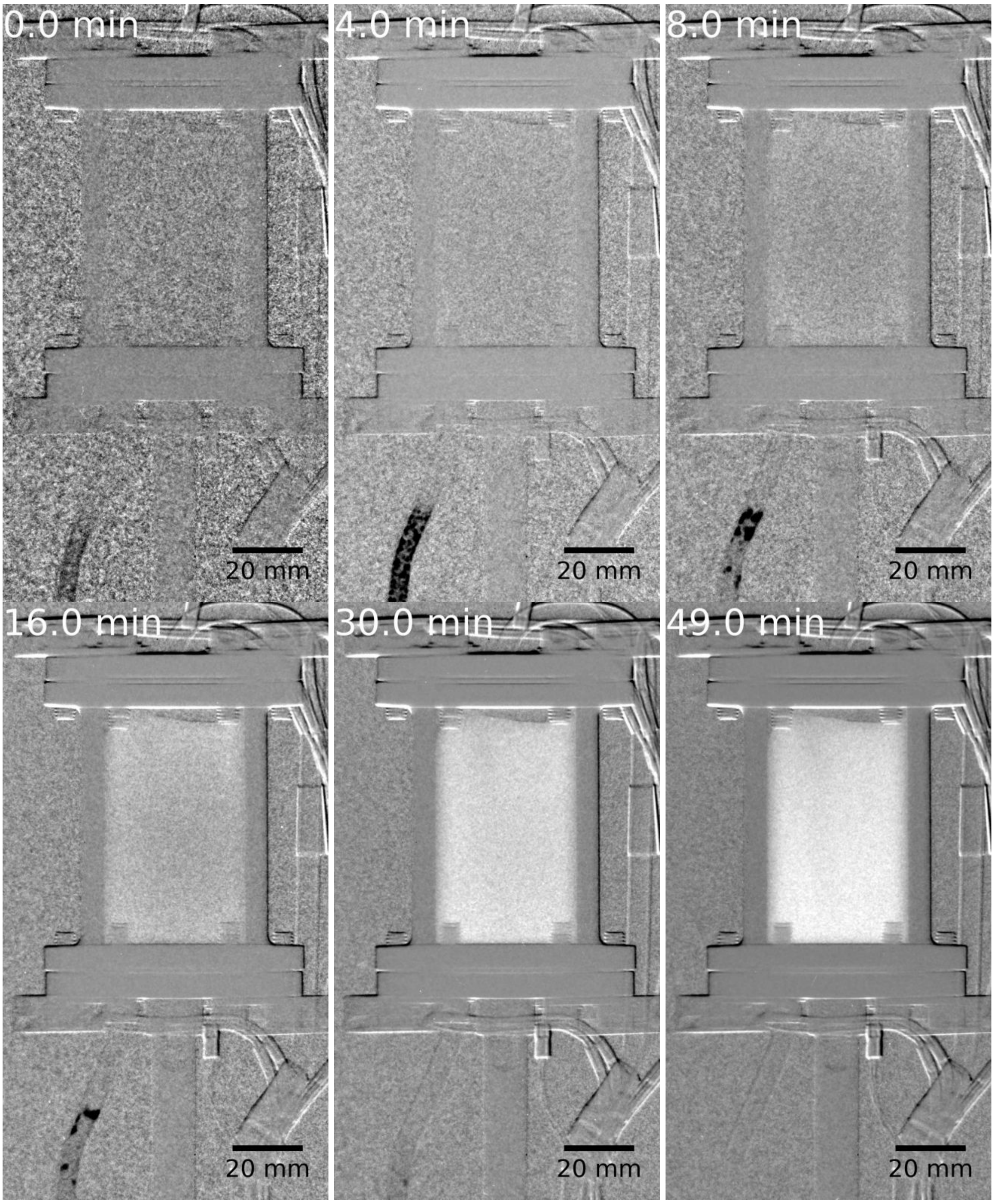

Figure S4. A series of difference frames taken during the drying of the catalyst. The cell was heated at 100 °C during hydrogen flow (10 ml min^{-1}) in helium (100 ml min^{-1}). The reference frame was taken prior to heating the cell and the timestamp is relative to the cell temperature reaching 100 °C. The condensing water in the unheated exhaust pipe is clearly visible as a dark, highly neutron attenuating signal. The catalyst bed is seen to become more neutron transmitting as the hydrogen content decreases with the loss of water, resulting in a bright region in the images.

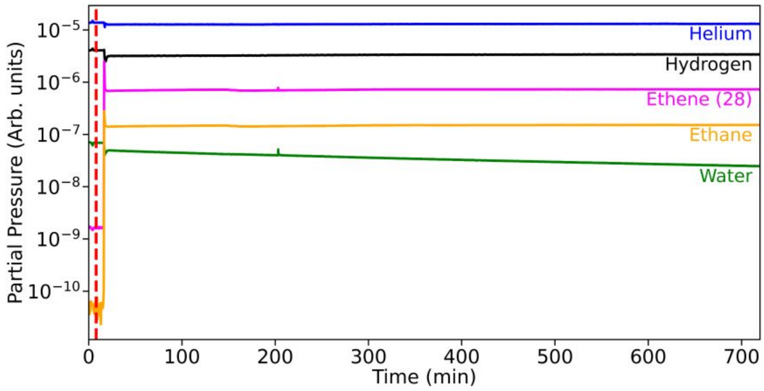

<u>Figure S5</u>. Mass spectrometric profile for the exit gas as a function of T-o-S during a period when the catalyst is maintained at 333 K and with imaging commencing in the presence of a He/H_2 co-feed (He = 100 ml min^{-1}, H_2 = 30 ml min^{-1}). At t = 10 min the feedstream is supplemented by the addition of ethene at a flow rate of 5 ml min^{-1}, representing a hydrogen:ethene ratio of 6:1 (v/v). This is an extended time version of Figure 6a in the main manuscript showing the slow decrease in the partial pressure of water signal in the mass spectrometer.

Increasing ethene flow in the absence of a hydrogen flow

To stress the hydrogenation system further, this section looks at the catalyst response to further increasing the ethene flow rate in the absence of hydrogen. At t = 9 min, the helium/ethene feedstream was switched from 100/3 ml min^{-1} to 100/12 ml min^{-1}. In this way the catalyst is experiencing an enhanced ethene load in the absence of hydrogen. Figure 10a shows that in addition to the ethene signal increasing, somewhat surprisingly, there is an increase in the ethane signal. However, the increase in ethane signal is almost 3 orders of magnitude lower than that seen for the ethene, and thus is thought to be attributed to a small amount of ethane impurity in the ethene itself (99.9% purity).

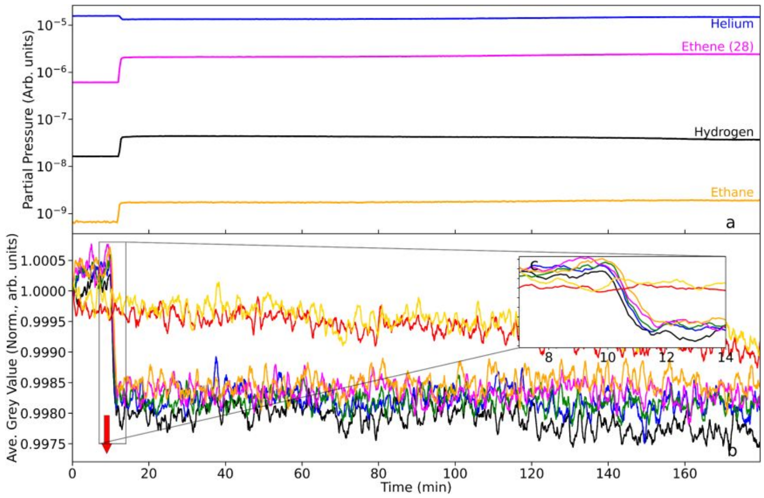

<u>Figure S6</u>. (a) Mass spectrometric profile for the exit gas as a function of T-o-S during a period when the catalyst is maintained at 60°C and with imaging commencing in the presence of a He/C_2H_4 co-feed (He = 100 ml min^{-1}, C_2H_4 = 5 ml min^{-1}), *i.e.* an absence of hydrogen. At t ~ 9 min the ethene flow rate is increased to 20 ml min^{-1}. (b) Corresponding average neutron grey values. The linkage between the colour of the neutron intensity plots and their respective location along the reactor are defined in Figure 1. (c) Zoomed inset showing the region around the perturbation in greater detail.

Figure S6 shows at the time when the ethene flow is increased (t = 9 min), all the five reactor RoIs show a sharp decrease in grey value of a small magnitude (δGV = 0.2%). Following that discrete drop, the hydrogen concentration level remains constant thereafter. The inset (Figure S6c) focuses in on the transition time and even at that close inspection, the reactor segments are bunched very close together but with a spatially resolved difference; the top of the catalyst bed reacting before the bottom. Thus, it can be deduced that only a small and brief concentration gradient is being introduced because of the perturbation, a situation which contrasts with that observed in, for example, Figure 2 (main manuscript).

A decrease in grey value corresponds to an increased presence of hydrogen-containing species that reduces the neutron transmission by a enhanced scattering. It is presumed that the small drop in neutron transmission arises as the four-fold increased partial pressure of ethene in the feedstream gas leads to a small increase in ethene adsorption on the catalyst and support. The magnitude of the drop in grey value evidenced in Figure 10b indicates that we are dealing with a small population of hydrogenous entities that remain bound to the catalyst surface under these conditions.
